# Identification of the Microbiota in Coconut Water, Kefir, Coconut Water Kefir and Coconut Water Kefir-Fermented Sourdough Using Culture-Dependent Techniques and Illumina–MiSeq Sequencing

**DOI:** 10.3390/microorganisms12050919

**Published:** 2024-04-30

**Authors:** Mansi Limbad, Noemi Gutierrez Maddox, Nazimah Hamid, Kevin Kantono, Colleen Higgins

**Affiliations:** Department of Food Science, Auckland University of Technology, Private Bag 92006, Auckland 1142, New Zealand; noemi.gutierrezmaddox@aut.ac.nz (N.G.M.); nazimah.hamid@aut.ac.nz (N.H.); kkantono@aut.ac.nz (K.K.); colleen.higgins@aut.ac.nz (C.H.)

**Keywords:** coconut water kefir fermented sourdough, MiSeq high-throughput Illumina sequencing, Sanger sequencing, species diversity, culture-dependent and culture-independent, titratable acidity, D-lactic acid, L-lactic acid

## Abstract

The principal objective of this study was to isolate and identify the microorganisms present in commercial kefir grains, a novel kefir-fermented coconut water (CWK) and a novel coconut water kefir-fermented sourdough using phenotypic identification and Sanger sequencing and examine the microbial diversity of CWK and CWK-fermented sourdough throughout the fermentation process using the MiSeq Illumina sequencing method. The phenotypic characterisation based on morphology identified ten isolates of LAB, five AAB and seven yeasts from kefir (K), CWK and CWK-fermented sourdough (CWKS). The results confirm the presence of the LAB species *Limosilactobacillus fermentum*, *Lactobacillus. plantarum*, *L. fusant*, *L. reuteri* and *L. kunkeei*; the AAB species *Acetobacter aceti*, *A. lovaniensis* and *A. pasteurianus*; and the yeast species *Candida kefyr*, *Rhodotorula mucilaginosa*, *Saccharomyces cerevisiae*, *C. guilliermondii* and *C. colliculosa*. To the best of our knowledge, the identification of Rhodotorula from kefir is being reported for the first time. This study provides important insights into the relative abundances of the microorganisms in CWKS. A decrease in pH and an increase in the titratable acidity for CWK- and CWK-fermented sourdough corresponded to the increase in D- and L-lactic acid production after 96 h of fermentation. Significant reductions in the pHs of CWK and CWKS were observed between 48 and 96 h of fermentation, indicating that the kefir microorganisms were able to sustain highly acidic environments. There was also increased production of L-lactic acid with fermentation, which was almost twice that of D-lactic acid in CWK.

## 1. Introduction

Sourdough, an ancient fermentation method, is celebrated for its intricate microbial community and distinctive flavour profile [1]. When incorporated as a substrate for fruit-based kefir, sourdough not only introduces depth of taste but also enhances the nutritional content and probiotic diversity of the beverage [2]. This synergistic combination offers a palatable and health-promoting alternative, enriching both the gastronomic experience and potential wellness benefits for consumers. The microbiota of sourdough is predominantly composed of lactic acid bacteria (LAB) and yeast species. Ascomycetous yeasts are exclusively detected in the sourdough ecosystem, attributed to their fermentation capability in comparison to basidiomycete yeasts or dimorphic ascomycetes [3,4]. Commonly identified yeasts in sourdough include *Saccharomyces cerevisiae*, *Candida colliculosa*, *Pichia kudriavzevii*, *C. humilis*, *Wickerhamomyces (W.) anomalus* and *S. exiguous* [3,4]. The stability of sourdough in bakeries, utilizing baker’s yeast, depends on the collaboration between the LAB, yeasts and baker’s yeast [4,5]. In laboratory-developed sourdough, *S. cerevisiae*, *W. anomalus* and *C. glabrata* dominate, with heterofermentative LAB, particularly lactobacilli, being predominant in stable sourdough ecosystems [3]. 

Approximately 60 *lactobacilli* species, including facultatively and obligately heterofermentative and obligately homofermentative types, have been isolated from various sourdough types. Obligately heterofermentative lactobacilli, like *L. sanfranciscensis*, *Limosilactobacillus fermentum* and *L. reuteri*, exhibit highly adapted carbohydrate metabolism, stress response, and amino acid assimilation, contributing to the sourdough fermentation process [6,7,8]. The diversity of the LAB in sourdough reflects their adaptability, with certain species consistently associated with sourdough, such as *L. sanfranciscensis* and *L. (par)alimentarius*, while others like *L. brevis* and *L. plantarum* are not only frequently found in sourdough but also isolated from other fermented foods.

Kefir grains, as symbiotic clusters of bacteria and yeast, hold significant importance in fermented food production due to their ability to produce lactic acid, which contributes to numerous health benefits including improved gut health and immune function [9]. The fermentation process mediated by kefir grains results in the synthesis of bioactive compounds and probiotics, enhancing the nutritional value and therapeutic potential of kefir [10]. Kefir, a diverse ecosystem, harbours numerous LAB, yeast, and acetic acid bacteria (AAB) species, contributing to its complexity [11,12,13]. Lactobacillus spp and Lactococcus spp constitute a substantial portion of the kefir microbiota, with lactobacilli eventually identified as the most dominant of the microorganisms in kefir grains [13,14,15,16]. Predominant bacteria in kefir grains include *L. kefiri*, *L. kefiranofaciens*, *L. paracasei*, *L. acidophilus*, *L. delbrueckii* and *L. plantarum*, along with Acetobacter spp, as integral components of kefir grains [17,18,19,20,21]. The identification and characterisation of microorganisms responsible for fermentation are crucial for understanding the diversity and relative abundances of microorganisms over time. The lactic acid production by kefir grains plays a pivotal role in conferring various health benefits to humans, including improved gut microbiota, enhanced immune response, and anti-inflammatory effects [22,23]. These benefits contribute to overall well-being and may aid in the management of conditions such as gastrointestinal disorders and metabolic syndrome.

Phenotypic identification has contributed to our understanding of physiological properties, while phylogenetic identification reflects natural relationships within the microbial population [24]. In this study, microbiota in coconut water (CW), kefir grains, coconut water fermented with kefir (CWK), and CWK-fermented sourdough (CWKS) were identified and characterised. This study aimed to understand microbial community dynamics during fermentation, analyzing changes in microbial profiles and relative abundances at different time points (T = 0, 24, 48, 72, and 96 h).

To determine microbial diversity in CWK-fermented sourdough, a combination of culture-dependent phenotypic identification methods and culture-independent sequencing methods, including Sanger sequencing and Illumina MiSeq sequencing, were employed [25]. The characterisation of microbial activity in kefir grains and their impacts on the pH, total titratable acidity (TTA), and D- and L-lactic acid production in coconut water and sourdough were also studied. 

## 2. Materials and methods

### 2.1. pH, D-Lactic Acid, L-Lactic Acid and Total Titratable Acidity

The pH of kefir powder was determined by weighing 1 g of kefir powder that was added to 10 mL of distilled water and blended. To measure the pH of dough samples, 10 g of sourdough sample was added to 100 mL distilled water [26] and blended. Readings for pH were taken using a digital pH meter (Eutech pH 700 m, Thermo Fisher Scientific Inc., Auckland, New Zealand) with a glass electrode (Electrode ECFC7252101B, Thermo Fisher Scientific Inc., New Zealand). Before measurement, the pH meter was calibrated with buffers (Thermo Fisher Scientific Inc., New Zealand) at pHs 4.0 and 7.0. The pH measurements were performed in triplicate for each sample (at 0, 24, 48, 72 and 96 h).

The concentrations of D- and L-Lactic acid concentrations were determined using the Megazyme lactic acid kit K-DLATE01/14 (Megazyme, Auckland, New Zealand), following the manufacturer’s instructions. Total titratable acidity was determined using the AACC International Method 02-31.01 (AACC, 1999), where 10 mL of coconut water kefir sample and 10 g sample of coconut water kefir sourdough sample mixed with 100 mL of distilled water (at 0, 24, 48, 72 and 96 h) were titrated against 0.1 M NaOH, with a pH of 8.5. 

### 2.2. Phenotypic Characterisation and Identification of Species

#### 2.2.1. Samples 

The samples used for microorganism identification were fresh young coconut water (Countdown, Auckland) kefir grain powder purchased from Body Ecology™, Auckland, New Zealand; and CWK-fermented sourdough. 

#### 2.2.2. Methods of Analysis

For identification in the coconut water, 1 mL of uninoculated coconut water (10-fold dilution), in triplicate, was spread on De Man, Rogosa and Sharpe agar medium (MRS); malt extract (ME) agar; and acetic acid bacteria (AAB) agar (DIFCO, Fort Richard Laboratories, Auckland). For kefir grains, bacteria were obtained from serial dilution plates using MRS agar, ME agar and AAB agar for isolation and identification. Microorganisms for CWK fermentation were obtained from 300 mL of coconut water incubated with kefir grains (1.5 g/L) at 30 °C for up to 96 h. Samples were taken in triplicate at 24 h time intervals. A CWK sample fermented at 48 h (300 mL) in a food-grade LabServ incubator (Thermo Fisher Scientific, New Zealand) was used for the preparation of sourdough. 

#### 2.2.3. Preparation of Samples

Sourdough (600 g) was prepared using CWK (300 mL), table salt (3 g) and canola oil (1.5 mL). In total, 1 g of sourdough sample was taken every 24 h of fermentation, which was suspended in 9 mL of deionised water using a stomacher (or a mechanical mixer). Next, 1 mL of this liquid sample, in triplicate, was spread on the MRS, AAB and ME agar (DIFCO, Fort Richard Laboratories, Auckland). Phenotypic characteristics of isolates from kefir, CWK and CWK-fermented sourdough were evaluated by determining the cell morphology, colonial morphology and cultural, biochemical and physiological characteristics. Single colonies that differed in these characteristics were picked from each plate and Gram-stained. Gram-positive microorganisms were further subdivided into spore formers and non-spore formers based on spore staining (Schaeffer–Fulton method with malachite green stain). Subsequent identification was performed using motility, and biochemical tests were performed according to Bergey’s Manual of Determinative Bacteriology [27] and API kits, respectively. Putative LAB isolates that were Gram-positive, non-spore forming and catalase-negative were further analysed via cultural, biochemical and physiological characterisation, as shown in Tables S1–S3. The sugar fermentation characteristics were determined using the API CHL 50 biochemical test kit (Biomérieux, Mediray Laboratories, Auckland, New Zeland). Putative acetic acid-producing bacteria were identified based on their growth on AAB agar and in AAB broth. The putative AAB isolates were phenotypically identified using Bergey’s Manual of Determinative Bacteriology [27] followed by biochemical identification, which was carried out using an API 20 E kit (Mediray, New Zealand). Additionally, Carr’s medium (30 g yeast extract, 20 g ethanol, 0.02 g bromocresol purple and 20 g agar per litre, with a pH of 5.5–6.0) was used to confirm the presence of Acetobacter. 

Yeast isolates were identified from kefir grains, CWK and CWKS. Serial dilution plates on ME agar were prepared from kefir grains and CWK, as described earlier. For CWKS, 1 g was suspended in 5 mL of deionised water and placed in a stomacher bag for homogenisation (Stomacher 400 Circulator, Thermofisher, New Zealand) for 3 cycles at 60 s each. This was followed by centrifugation for 5 min at 5000 g. Next, 1 mL of the supernatant was used for 10-fold serial dilutions, which were plated on ME agar. The ME cultures were incubated at 29 °C for 48–72 h. Colonies that were non-shiny and bigger than pin-point colonies were selected and considered putative yeast colonies. These were purified using five-phase streak plating on ME agar. The API kit ID 20C AUX (Mediray, New Zealand) was used to identify the putative yeast isolates, as per the manufacturer’s instructions. The positive or negative results (after incubation) were recorded as (+) or (−), respectively, and the isolates were identified using the API-web (AB bioMérieux). 

### 2.3. Identification Using Sanger Sequencing Method

All the LAB and acetic acid bacterial isolates identified previously were grown in triplicates in MRS broth at 30 °C for 48 h. The broths were centrifuged at 5000 rpm for 10 min (Heraeus Labofuge 200; Thermo Scientific). For genomic DNA isolation, cell pellets were re-suspended in double-distilled water and microwaved for 30 s at 150 °C (Living and Co., Auckland, New Zealand). This was followed by centrifugation at 1000 rpm for 5 min at room temperature (Heraeus Labofuge 200; Thermo Scientific). The supernatant (about 5 µL) was used in a polymerase chain reaction (PCR) with 5 pmol each of the forward and reverse primers, PCR1 forward (5′ TCGTCGGCAGCGTCAGATGTGTATAAGAGACAGCCTACGGGNGGCWGCAG 3′) and PCR1 reverse (5′ GTCTCGTGGGCTCGGAGATGTGTATAAGAGACAGGA CTACHVGGGTATCTAATCC 3′), with KAPA HiFi Hotstart Readymix (Kapa Biosystems, Wilmington, MA, USA). The following thermocycling parameters were used: 95 °C for 3 min, 25 cycles of 95 °C for 30 s, 55 °C for 30 s, 72 °C for 30 s, 72 °C for 5 min, and a holding temperature of 15 °C. PCRs were carried out using an Eppendorf vapoproject AG 223331 PCR machine (Hamburg, Germany).

The presence and quality of the 500 bp amplicon were confirmed using a 2100 bioanalyser instrument (Agilent Technologies, Santa Clara, CA, USA) with a DNA 100 chip. The amplicons were indexed using a Nextera XT index kit with an additional eight PCR amplification cycles using the same PCR conditions as above, purified and size-selected using AMPure XP beads (Beckman-Coulter, Brea, CA, USA) by Auckland Genomics (The University of Auckland, Auckland, New Zealand). DNA sequences were curated using Geneious v5.6 (https://www.geneious.com, 21 November 2019)). Sequences were identified via BLASTn analysis (https://blast.ncbi.nlm.nih.gov/Blast.cgi, accessed on 22 December 2019). 

### 2.4. MiSeq High-Throughput Sequencing Methods (Illumina Sequencing) 

DNA was extracted from 1 mL of coconut water and 1 g of kefir powder each and dissolved in 5 mL of phosphate buffer saline. For CWK, 1.5 g/L of kefir grains was added to 300 mL coconut water, and samples were collected following 0, 24, 48, 72, and 96 h of fermentation and stored at −80 °C. The coconut water kefir sample fermented for 48 h was used as an inoculum to prepare coconut water kefir sourdough. Sourdough was proofed at 30 °C, with 60% humidity. Pizza-wedged slices were sampled from coconut water kefir sourdough at 0 h, 24 h, 48 h, 72 h and 96 h and stored at −80 °C. 

Dough samples were mixed in a 1:1 ratio with phosphate-buffered saline with a pH of 7.4 in a homogeniser (L5M-A Laboratory Mixer, Silverson^®^, Chesham, UK) and allowed to digest for 1 h to help release microbial DNA from the wheat matrix of the dough. DNA was isolated from triplicate samples using a PowerFood DNA Isolation kit (Mo Bio Laboratories, Carlsbad, CA, USA) following the manufacturer’s protocol. To improve cell lysis, samples were incubated at 65 °C for 30 min before vortexing. An extraction blank was prepared with water, which served the purpose of the blank used in this study. 

Bacteria and yeasts were targeted for identification since they are the most abundant microorganisms in sourdough [28]. PCRs targeting the V3–V4 regions of the bacterial and archaeal 16S rRNA gene were carried out using the primers described above in Section 2.2, with KAPA HiFi Hotstart Readymix (Kapa Biosystems, Wilmington, MA, USA). The confirmation of the PCR products and indexing were carried out as described above in Section 2.2. 

Amplicon library preparation was performed according to the recommended protocols (Illumina Demonstrated Protocol: 16S Metagenomic Sequencing Library Preparation—Amplicon, Clean-Up and Index). Up to 96 uniquely indexed libraries were pooled per sequencing run, which was performed on an Illumina MiSeq using 300-cycle V2 chemistry (150 bp paired-end reads) following the manufacturer’s recommendations. No-template and extraction reagent blank (EXB) controls were also sequenced to establish background bacterial populations. The method of analysis for Illumina output followed that published by Archer et al. (2019) [29]. 

### 2.5. Bioinformatics Analysis 

The microbial composition of each sample was examined by processing the raw sequence reads using USEARCH v 8.0.1623 [30]. The quality of these raw sequencing reads was analysed using the USEARCH tool to remove anomalous sequences. Paired-end reads with lengths outside the 200–500 bp range or exceeding six homopolymers were removed by Mothur v1.36.1 [31]. Next, the sequences were subjected to *Q*-score filtering to remove reads with maximum expected error > 1. Singleton reads were then removed. Representative sequences of the operational taxonomic units (OTUs) were taxonomically assigned using the RDP classifier implemented in QIIME v1.9.1 [32]. Greengenes release 13_8 was used as the reference taxonomic database [33]. 

## 3. Results and Discussion

### 3.1. pH, D-Lactic Acid, L-Lactic Acid and Total Titratable Acidity Determination

The pH, D-lactic acid, L-Lactic acid and total titratable acidity (TTA) of the coconut water kefir-fermented sourdough at 0, 24, 48, 72 and 96 h incubation at 30 °C are summarised in Table 1. 

D-lactic acid, L-lactic acid, total titratable acidity (TTA) and pH were determined for kefir, coconut water, CWK and CWK-fermented sourdough, as shown in Table 1. Kefir grains had a pH of 4.56 ± 0.6, TTA of 6.14 ± 0.12, D-lactic acid concentration of 0.16 ± 0.03 g/L and L-lactic concentration of 0.55 ± 0.05 g/L. Ribeiro et al. (2020) [34] and Irigoyen (2005) [35] reported that the pH of kefir was between 4.2 and 4.6, similar to this study, and corresponded to the overall titratable acidity values of the kefir grains. The lactic acid concentration of kefir grains in this study was, however, lower than that reported by Ribeiro et al. (2020) [34] for milk kefir grains, which had a lactic acid concentration of 4 mg/g. This could be due to the fact that water kefir grains were used in this study instead of milk kefir grains.

Coconut water had a pH of 5.65 ± 0.21, TTA of 5.34 ± 0.18, D-lactic acid concentration of 1.68 ± 0.54 g/L and L-lactic concentration of 2.55 ± 0.33 g/L, which was almost double the concentration of D-Lactic acid. Kannangara et al. (2018) [36] reported that coconut water has a pH between 5.39 and 6.32 and a titratable acidity of 5.3, similar to this study. 

The LAB and yeast that grew during the fermentation of coconut water increased the lactic acid concentration of the medium through carbohydrate metabolism. The production of L- and D-lactic acid during the fermentation influences the overall quality of the product. The presence of D-lactic acid is undesirable as it forms non-metabolisable constituents, which may accumulate in the blood [37]. The concentration of L-lactic acid was almost double the concentration of D-lactic acid, as observed in Table 1. L-lactic acid was by far the highest in CWK fermented for 96 h compared to that produced at 72 h, 48 h and 24 h (*p* < 0.05). The highest concentration of D-lactic acid was also produced after 96 h of fermentation. The next highest concentrations were obtained at 72 h, 48 h and 24 h. The increase in lactic acid can be explained by the utilisation of carbohydrates in the coconut water during the fermentation of coconut water with kefir. This agrees with the study carried out by Liu and Lin (2000) [38] on milk kefir grains, which reported an increase in the overall lactic acid content of milk kefir grains with time. There is some physiological importance in terms of type of lactic acid produced in fermented milk kefir (Farnworth, 2008) [39]. Two stereoisomers, L- and D-isomers, are formed in kefir grains through the action of homofermentative and heterofermentative microorganisms. In the gastrointestinal tract, both D- and L-Lactic acid isomers are completely absorbed. However, their proportions for the production of glucose and glycogen differ hugely. L-lactic acid is completely converted to glycogen at a rapid metabolic rate, whereas D-lactic acid is metabolised at a much slower rate and further excreted out of the body with urine (Uniacke-Lowe, 2011) [40]. It has been reported that kefir grains contain mainly L-lactic acid that varies greatly with the kefir growth medium. A higher overall concentration of L-lactic acid than D-lactic acid was found in this study in CWK fermented for 96 h. Similarly, at the end of milk kefir fermentation, the concentration of L-lactic acid was much lower than the concentration of D-lactic acid [39,41,42]. There was significant production of D-Lactic acid in the CWK fermented sourdough of 10.26 ± 0.67 when compared the production of L-Lactic acid, which was less than half 3.17 ± 0.01.

During fermentation, glucose and sucrose were metabolised to produce acids, which correlated with a drop in the pH (Table 1) and an increase in the overall acidity of the CWK medium. This acidity, measured as the total titratable acidity (TTA) in Table 1, increased significantly with time (*p* < 0.05). CWK fermented at 96 h had by far the highest amount of TTA (11.71 ± 0.02), followed by fermentation at 72 h, which implies a link to the fermentation time. A big increase in total titratable acidity (up to 1% at the end of fermentation) was previously reported for milk kefir grains by Chen et al. (2009) [43] that was accompanied with a decrease in pH and an increase in lactic acid and the production of other organic acids [43]. The transformation of sugars to lactic acid and other organic acids by the LAB is an important step during fermentation that influences the pH and acidity of fermented products. 

Overall, a time-dependent pattern could be identified with fermentation times at 96, 72 and 48 h of incubation at 30 °C that had significantly higher values for D-lactic acid, L-lactic acid, pH and TTA compared to the 24 and 0 h fermentation periods. These results are consistent with results from several other studies [44,45,46,47,48,49]. Low pH values are suitable for the growth of acid-tolerant lactobacilli, whereas higher values select for species of *Enterococcus*, *Lactococcus*, *Leuconostoc*, *Pediococcus* and *Weissella* [44,45,46,47,48,49]. The low pH values in the dough indicated the acidification of the dough due to acetic acid and lactic acid production by acid-tolerant LAB species. Therefore, high D-/L-lactic acid content was found in the dough at the end of the fermentation period, which corresponded to low pH and high TTA values. 

A prolonged fermentation time in kefir enhances the stability of lactic acid bacteria (LAB), promoting higher LAB counts and diversity due to prolonged metabolic activity [50]. This increased stability may improve the probiotic potential and sensory characteristics of the final kefir product.

### 3.2. Identification 

Microbial growth was observed in the MRS, AAB and ME media for kefir grains, coconut water fermented with kefir and sourdough prepared with CWK at different time intervals (0, 24, 48, 72 and 96 h). The phenotypic characterisation based on the morphology identified ten isolates of LAB, five isolates of AAB, and seven yeasts from kefir (K), CWK and CWK-fermented sourdough (CWKS). Upon the completion of the phenotypic screening, only 13 isolates of all LAB, yeasts and AAB were confirmed. 

All ten preliminary LAB isolates were Gram-positive, non-motile, catalase-negative and non-spore-forming, typical of LAB [51]. They occurred in short rods, singly, in pairs or in short chains. All isolates tolerated bile salt at 0.1–2% and grew at 15 °C but not at 45 °C. No growth was observed for any isolate at 10% NaCl, whereas at 6.5% NaCl, growth was observed for all except one isolate (Supplementary Table S1). All five preliminary AAB isolates were white–cream in colour and had opaque colonies with smooth surfaces, as expected for AAB. They were Gram-negative and catalase-positive. The cells were bacilliform, and further physiological and biochemical tests showed that they belonged to the genus *Acetobacter* (Supplementary Table S2). Seven putative isolates of yeast had white- and cream-coloured colonies with irregular surface appearances. The morphology of these cells was oval and showed evidence of budding. The cell morphologies of these isolates are presented in Supplementary Table S3. The presumptive isolates were further characterised using growth at different temperatures (10 °C, 30 °C and 45 °C), on media with different pH and salt conditions, and in different sugar media (using API kits, according to the manufacturer’s instructions). 

Supplementary Table S1 shows that biochemical testing identified *Limosilactobacillus fermentum*, *L. plantarum*, *L. fusant* and *L. reuteri* in kefir, CWK and/or CWKS.

Overall, out of the 22 isolates, five species of LAB (Supplementary Table S1), three species of AAB (Supplementary Table S2) and five species of yeast (Supplementary Table S3) were identified using morphological, physiological and biochemical methods. Possibly, some of the 22 isolates were identical to each other. The species identified were *Limosilactobacillus fermentum*, *Lactobacillus. plantarum*, *L. fusant*, *L. reuteri*, *L. kunkeei*, *A. aceti*, *A. lovaniensis*, *A. pasteurianus*, *C. kefyr*, *R. mucilaginosa*, *S. cerevisiae*, *C.guilliermondii* and *C. colliculosa*.

All the identified LAB utilised galactose, glucose and sucrose and, thus, were likely the main organisms responsible for the fermentation in CWK samples. Such similar characterisation for kefir has been carried out by Diosma and Romanin et al. (2014) [52], Witthuhn, et al. (2005) [53] and Zanirati et al. (2015) [54]. Their studies show a similar sugar utilisation profile for all the identified LAB species. The microbial composition may vary according to the kefir’s origin, the substrate used in the fermentation process and the culture maintenance methods. Tibetan kefir, which is used in China, is composed of *Lactobacillus*, *Lactococcus* and yeast [55]. Sun et al. (2015) [56] reported the presence of over 200 species of LAB in a traditionally fermented sourdough and highlighted the commercially important LAB species. Some of the dominant species identified by them were *L. crustorum*, *L. parabrevis*, *L. pobuzihii* and *L. selangorensis*. Various LAB species have also been identified in milk kefir; some that dominated were *Leuconostoc mesenteroides*, *L. skefiri,* and *L. kefiranofaciens* (Zanirati, Abatemarco, et al. 2015) [54]. 

The presence of AAB in kefir has been reported previously by Garrote [57], Abraham and De Antoni (2002). AAB such as *A. aceti* have been identified in Tibetan kefir, which has been previously reported by Gao et al., (2012) [58]. To the best of our knowledge, this is the first study reporting the presence of *A. lovaniensis* and *A. pasteurianus* in CWK and CWKS. 

The sequence of 16S rDNA helped to confirm the identity of the LAB and AAB identified (Supplementary Tables S4 and S5, Supplementary File S1). The partial sequences for both LAB and AAB are provided in Supplementary Table S5, which were the final output of a completed alignment of forward and reverse sequences obtained. The sequences were aligned using Geneious^®^ 11.1.4 after manually removing the noise on both ends of the forward and reverse sequences. These partial sequences were confirmed using NCBI BLASTn and the identification of species for the organisms was selected for the one with the highest maximum score and the lowest E-value (for high significance). *Limosilactobacillus fermentum* strain CAU6479 16S ribosomal RNA gene, partial sequence (100); *L. plantarum* strain RS66X 16S ribosomal RNA gene, partial sequence (100); *L. Fusant* XU1 16S ribosomal RNA gene, partial sequence (99); *L. reuteri* DSM 20016, 16S ribosomal RNA gene, partial sequence (100); *L. kunkeei* strain H14_2_1BCO2 16S ribosomal RNA gene, partial sequence (100); *A. aceti* strain W1 16S ribosomal RNA gene, partial sequence (100); *A. lovaniensis* strain NBRC 13753 16S ribosomal RNA, partial sequence (100); and *A. pasteurianus* strain bh12 16S ribosomal RNA gene, partial sequence (100) were identified. 

Yeasts were identified according to the criteria of Kurtzman, Fell, and Boekhout (2011) [59]. Five species belonging to three genera were identified (Supplementary Table S1). The Food and Agriculture Organization of the United Nations has stated that kefir possesses lactose-fermenting yeasts (*C. kefyr*) and non-lactose fermenting yeasts (*R. mucilaginosa*, *S. cerevisiae*, *C. guilliermondii* and *C. colliculosa*); however, the yeast composition has not yet been fully defined, although other authors have found additional species [60]. *Candida*, *Saccharomyces* and *Rhodotorula* were isolated in the present work from the CWK and sourdough fermented with CWK. To the best of our knowledge, identification of *Rhodotorula* from kefir is being reported for the first time. *Candida* and *Saccharomyces* are among the yeasts identified in kefir grains from China, Germany and the United States [61]. The composition of kefir microflora appears to strongly depend on the origin of the grains, the local conditions of culture, and the storage and elaboration processes, which could explain why such diverse microflora has been identified from New Zealand kefir grains [57]. The only yeasts identified in kefir so far are *Kluyveromyces*, *Torulaspora*, *Saccharomyces*, *Candida*, *Pichia*, *Kazachastania*, *Lachanceae*, *Yarrowia* and *Mycotorula* (Kolakowski & Ozimkiewicz, 2012 [62]; Leite et al., 2012; Magalhães et al., 2010 [63]; Simova et al., 2002 [64]; Wang & Chen et al., 2008 [20]). 

Most of the LAB and AAB that have been identified in this study, such as *Limosilactobacillus fermentum*, *L. plantarum*, *L. fusant*, *L. reuteri*, *L. kunkeei*, *A. aceti*, *A. lovaniensis* and *A. pasteurianus*, have been identified and reported for kefir in previous studies. Studies carried out by Sakamoto and Tanaka et al. (2011) [65] and Miyashita et al. (2012) [66] identified *L. plantarum*, *L. rueteri* and *Limosilactobacillus fermentum* from sourdough by using 16s rRNA gene-sequencing- and pyrosequencing-based methods. *A. aceti* and *A. pasteurianus* have been previously used in the preparation of sourdough in a study carried out by De Pauw (2018) [67]. The identification of *Acetobacter aceti*, *Acetobacter lovaniensis* and *Acetobacter pasteurianus* has been reported in kefir and kefir beverages by Rattray and O’Connell (2011), Rosa et al. (2017) [13], Garofalo et al. (2015) [68,69], Prado et al. (2015) [70], Bourrie et al. (2016) [71] and Walsh et al. (2016) [16]. In a separate study carried out on Brazilian kefir grains, Magalhães et al. (2011) [72] reported that LAB and yeast species such as *L. paracasei*, *L. parabuchneri*, *L. casei*, *L. kefiri*, *Lactococcus lactis* and *S. cerevisiae* have been identified from kefir using 16s rRNA gene-sequencing methods. 

A common sourdough LAB, *L. sanfranciscensis*, was not identified in this study, which could be due to the sourdough formulated in this study being a laboratory type 2 fermented sourdough prepared using kefir powder. The coconut water was used as a growth medium for fermenting the kefir grains. Perhaps, due to lack of appropriate nutrients, the *L. sanfranciscensis* strain was not favoured to grow. The absence of this species might also be due to the presence of competitive bacterial strains. The complexity and stability of the sourdough microbiota depend on a number of determinants, which include environmental microbiota (e.g., microbiota of flour and other ingredients and house microbiota) and their potential metabolic activities (e.g., cofactor regeneration capability and energy synthesis from various sources), as well as technology parameters (e.g., the chemical and enzyme composition of the flour, leavening temperature, pH and redox potential, dough yield, and number and length of sourdough refreshments) [6,7,73,74,75], which explains why the D-Lactic acid was higher in CWK-fermented sourdough compared to L-Lactic acid. Bessmeltseva et al. (2014) [76], Ercolini et al. (2013) [28], Minervini et al. (2015) [77], Rizzello et al. (2015) [78] and Van der Meulen et al. (2007) [79] have reported that *L. sanfranciscensis* is generally absent in laboratory-made sourdoughs started with flour only.

### 3.3. Relative Abundance of Microorganisms Using Miseq High-Throughput Sequencing Method

**Figure 1:**
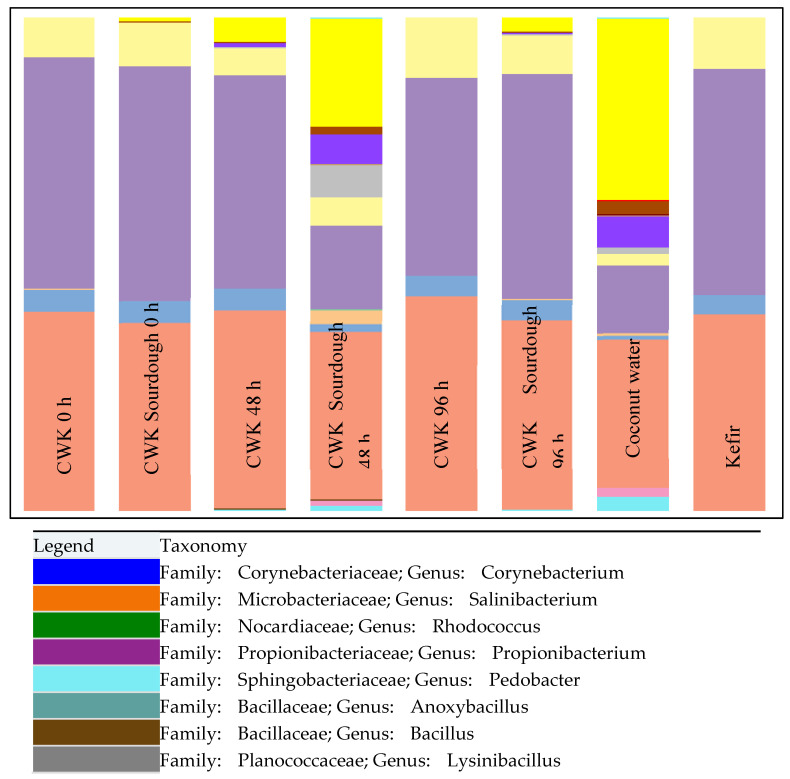

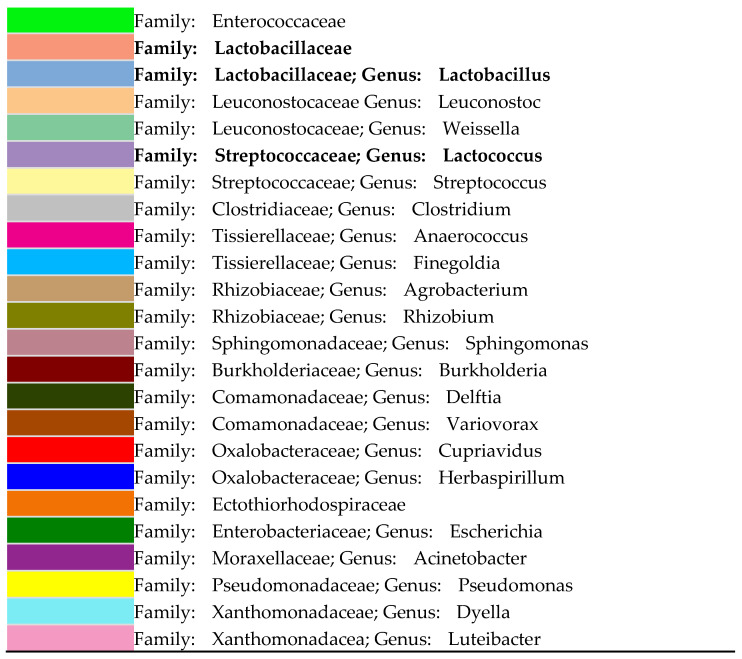
Stacked bar graph representing the bacterial compositions of CWK-fermented sourdough (at times, T = 0, 48 and 96 h), fermented CWK (at times, T = 0, 48 and 96 h), coconut water and kefir determined via the MiSeq Illumina sequencing method.

Community analysis (Figure 1) showed that the LAB were dominant throughout the duration of fermentation of both CWK and CWK-fermented sourdough. 

Analysis of the relative abundances of the different microorganisms showed that amongst all samples of kefir, coconut water, CWK, and CWK-fermented sourdough at all time points between 0 and 96 h, eight LAB species (*L. lactis*, *L. plantarum*, *L. rhamnosus*, *L. delbrueckii*, *L. paracasei*, *L. brantae*, *L. reuteri* and *L. kunkeei*) were highly abundant in all the samples. The most common types of LAB that have been previously identified for laboratory type 2 sourdough were *L. sanfranciscensis*, *L. brevis*, *Limosilactobacillus fermentum*, *L. reuteri*, *L. pontis*, *L. panis* and *Weissella* spp. [3,7,45,80,81,82,83]. Two out of the above-mentioned LAB, which were *Limosilactobacillus fermentum* and *L. reuteri*, were identified in the present study, which were both obligately heterofermentative microorganisms. *L. plantarum*, which has also been identified in this study, is a facultatively heterofermentative LAB, which is usually isolated and identified from continuously backslopped sourdough [84]. 

**Figure 2:**
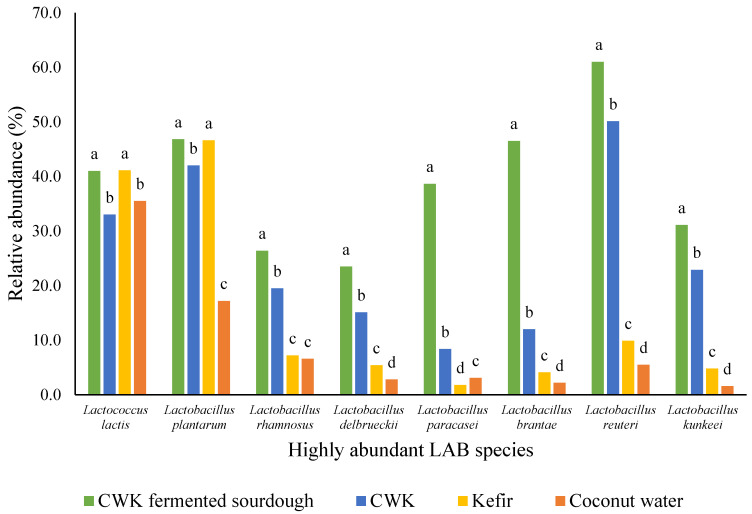
The relative abundances of 8 LAB species for CWK fermented sourdough, CWK, CW and kefir. These 8 LAB species are the highly dominating species across all the samples. Letters a–d represent significant changes for each isolate for CKW-fermented sourdough, CWK, kefir and coconut water.

The analysis of metagenomic data (Figure 2) reveals that all eight LAB species investigated exhibited the highest relative abundance in coconut water kefir-fermented sourdough, followed by coconut water kefir and kefir. The observed differences in LAB species abundance among the three fermented products can be attributed to variations in substrate availability during fermentation. Sourdough, being a solid matrix rich in carbohydrates and nutrients, likely provides more abundant and diverse substrates for microbial growth compared to liquid-based coconut water kefir and kefir [85,86]. This enhanced substrate availability in sourdough may promote higher microbial activity and species diversity, leading to increased LAB abundance.

*L. lactis and L. plantarum* were highly abundant in CWK-fermented sourdough (Figure 2). For these two species, the relative abundance was equivalent in CWK and Kefir. *L. plantarum* and *L. lactis* are commonly found in kefir grains and sourdough (Farnworth & Mainville, 2008; Witthuhn, Schoeman, & Britz, 2005) [39,53]. The detection of *L. reuteri* in kefir has been reported by Bourrie, Willing and Cotter (2016) [71] and Rosa et al. (2017) [13]. These species have also been identified in sourdough during fermentation, as reported by Huys et al. (2013) [3] and Minervini et al. (2014) [87].

A study carried out by Chen [88], Wang and Chen (2008) [89] reported the presence of various LAB species in kefir, including *L. rhamnosus*, *L. delbruecki* and *L. paracasei*, using a PCR–denaturing gradient gel electrophoresis (PCR-DGGE) method. 

It is interesting to note that five out of the eight species identified using the Illumina sequencing method, namely *L. lactis*, *L. rhamnosus*, *L. delbrueckii*, *L. paracasei* and *L. brantae*, were not identified using culture-dependent phenotypic identification methods. This might be due to either similarities between colonies, the method of isolation or microorganisms with large population sizes in the sample giving greater amounts of template DNA, leading to a higher likelihood of detection via Illumina sequencing compared to the microbial identification methods [90].

## 4. General Discussion

The objective of this study was to identify and characterise the microorganisms present in the kefir grains, fermented coconut water kefir (CWK) and sourdough fermented with coconut water kefir grains (CWKS). The results obtained in this study showed that the combined methods of phenotypic identification, biochemical tests and Sanger sequencing were able to identify five LAB species, three AAB species and five yeast species from kefir, CWK and CWKS. The species identified were *Limosilactobacillus fermentum*, *L. plantarum*, *L. fusant*, *L. reuteri*, *Lactobacillus kunkeei*, *Acetobacter aceti*, *A. lovaniensis*, *A. pasteurianus*, *Candida kefyr*, *Rhodotorula mucilaginosa*, *Saccharomyces cerevisiae*, *C. guilliermondii* and *C. colliculosa*. Some of these are common sourdough microflora such as *Lactobacillus fermentum*, *Lactobacillus plantarum*, *L. reuteri*, *Acetobacter aceti* and *Saccharomyces cerevisiae*, and the rest have been recently identified. 

Next, the prevalent microbial ecology of the CWKS, CWK, kefir and coconut water were determined using the MiSeq Illumina sequencing method. The results provided great insights into the relative abundances of the microorganisms present in each of the samples at a given time. The results demonstrated that LAB are present at a higher abundance compared to the rest of the microorganisms in each of the kefir, CWK and CWKS samples. The microorganisms present at higher incubation times between 48 and 96 h suggested that the microorganisms present in those samples were able to sustain highly acidic environments compared to those present during the earlier fermentation periods between 0 and 48 h. The succession of strains and species during sourdough propagation could affect the functional attributes of sourdough, and this, in turn, would influence the characteristics of the final product.

Additional LAB species were revealed to be present in kefir, CWK and CWKS samples using the Illumina sequencing method, which could not be identified using phenotypic or biochemical tests. Therefore, the phylogenetic characterisation of these sample using Illumina sequencing may be more beneficial in determining the microbial species present in them. 

Furthermore, it should be noted that *lactococci* were identified using SEM and Illumina techniques but were not identified using the phenotypic identification using microbial methods, which could be because of the disadvantages of using the technique. The most probable reasons could be that the *lactococci* could be lost during the process of transfer or storage due to its complex nature and the use of multiple biochemical media.

## 5. Conclusions

To summarise, high concentrations of L-lactic acid and D-lactic acid and pHs and TTAs were obtained for CWK and CWK-fermented sourdough at 96 h. There was increased production of L-lactic acid with fermentation, which was almost twice that of D-lactic acid in CWK. This, in turn, led to a decrease in the pHs of CWK and CWK-fermented sourdough after 96 h of incubation. The results obtained for the identification and characterisation using combined phenotypic and Sanger sequencing methods confirmed the species-level identification for LAB and AAB in CWK and sourdough fermented with coconut water kefir. The diversity of microorganisms in the sourdough fermented with CWK and CWK (fermented up to 96 h) was studied over time, and these results provided important insights into the relative abundances of the microorganisms present at a given time point. Eight LAB species were highly abundant across coconut water, kefir, CWK and CWK-fermented sourdough samples, which were *L. lactis*, *L. plantarum*, *L. rhamnosus*, *L. delbrueckii*, *L. paracasei*, *L. brantae*, *L. reuteri* and *L. kunkeei*. Additionally, the coconut water and kefir microflora were sequenced, with the majority belonging to the Lactobacillaceae family. 

## Figures and Tables

**Table 1:** The concentrations of L-Lactic acid and D-Lactic acid and the changes in pHs and TTAs estimated for kefir, coconut water, CWK and CWK-fermented sourdough at 30 °C between the 0 and 96 h time intervals. All the values for each experimental set represent the mean of triplicate readings.

Analysis	Kefir	Coconut Water	CWK					CWK-Fermented Sourdough				
			0 h	24 h	48 h	72 h	96 h	0 h	24 h	48 h	72 h	96 h
**L-Lactic acid (g/L)**	**0.55 ± 0.05 c**	2.55 ± 0.33 c	2.29 ± 0.02 E,c	2.59 ± 0.06 D,c	3.03 ± 0.05 C,c	4.85 ± 0.03 B,b	5.56 ± 0.1 A,b	2.1 ± 0.01 E,c	3.92 ± 0.07 D,d	4.34 ± 0.05 C,c	6.25 ± 0.37 B,c	8.08 ± 0.28 A,c
**D-Lactic acid (g/L)**	0.16 ± 0.03 d	1.68 ± 0.54 d	1.52 ± 0.05 E,d	2.07 ± 0.02 D,d	2.69 ± 0.04 C,d	2.94 ± 0.04 B,d	3.17 ± 0.01 A,d	2.14 ± 0.05 E,c	4.18 ± 0.14 D,c	6.6 ± 0.41 C,b	7.79 ± 0.35 B,b	10.26 ± 0.67 A,a
**pH**	4.56 ± 0.66 a	5.65 ± 0.21 a	5.55 ± 0.01 A,a	5.35 ± 0.01 B,b	4.29 ± 0.01 C,b	4.13 ± 0.01 D,c	3.81 ± 0.05 E,c	5.55 ± 0.09 A,a	4.71 ± 0.33 B,b	4.29 ± 0.22 C,c	3.99 ± 0.14 D,d	3.94 ± 0.14 E,d
**TTA (0.1 M NaOH)**	6.14 ± 0.12 b	5.34 ± 0.18 b	5.21 ± 0.02 E,b	6.50 ± 0.04 D,a	8.85 ± 0.04 C,a	10.26 ± 0.05 B,a	11.71 ± 0.02 A,a	5.18 ± 0.04 E,b	6.94 ± 0.06 D,a	7.3 ± 0.93 C,a	9.44 ± 0.26 B,a	9.92 ± 1.51 A,b
												
**F-value**	100,457.100	151.866	13,672.771	1285.290	60,546.750	23,335.185	59,578.161	2347.616	148.945	671.822	1015.578	8019.417
***p*-value**	<0.0001	<0.0001	<0.0001	<0.0001	<0.0001	<0.0001	<0.0001	<0.0001	<0.0001	<0.0001	<0.0001	<0.0001
**Significant**	Yes	Yes	Yes	Yes	Yes	Yes	Yes	Yes	Yes	Yes	Yes	Yes

A, B, C, D, E and F = Different letters within the same row (different fermentation times of 0 h, 24 h, 48h, 72h and 96 h for the same type of analysis) differ significantly using Fisher’s least significant difference (*p* < 0.05). a, b, and c: Different letters within the same column (different analysis for the same time interval) differ significantly using Fisher’s least significant difference (*p* < 0.05).

## Data Availability

The original contributions presented in the study are included in the article/Supplementary Materials, further inquiries can be directed to the corresponding author/s.

## Supplementary Materials

The following supporting information can be downloaded at: https://www.mdpi.com/article/10.3390/microorganisms12050919/s1, Table S1: Results of cell and colony morphology, physiological and biochemical tests performed on LAB isolates. Isolates identified from multiple sources such as kefir (K), CWK and/or CWK-fermented sourdough (CWKS) have been labelled accordingly; Table S2: Results of cell and colony morphological, physiological and biochemical tests performed on AAB isolates. Isolates identified from multiple sources such as kefir (K), CWK and/or CWK-fermented sourdough (CWKS) have been labelled accordingly; Table S3: Results of cell and colony morphology, physiological and biochemical tests performed on yeast isolates. Isolates identified from multiple sources such as kefir (K), CWK and/or CWK-fermented sourdough (CWKS), have been labelled accordingly; Table S4: Sanger sequencing of each isolate of the LAB and AAB species; File S1: Sanger sequencing output: 16s rDNA partial sequences for LAB and AAB.

## Abbreviations

LAB: lactic acid bacteria; AAB, acetic acid bacteria; CWK, coconut water kefir; CWKS, fermented sourdough (CWKS); rDNA, ribosomal DNA; PCR, polymerase chain reaction; DGGE, denaturing gradient gel electrophoresis; MRS, De Man, Rogosa and Sharpe agar medium; ME, malt extract agar; AAB, acetic acid bacteria agar; TTA, total titratable acidity.
