# Supplementary material for: Identification of the Microbiota in Coconut Water, Kefir, Coconut Water Kefir and Coconut Water Kefir-Fermented Sourdough Using Culture-Dependent Techniques and Illumina–MiSeq Sequencing
Source: Microorganisms. 2024 Apr 30;12(5):919. doi: 10.3390/microorganisms12050919 (PMC11124093; doi:10.3390/microorganisms12050919)
Supplement: Supplementary file 1 [file microorganisms-12-00919-s001.zip › microorganisms-2955548-supplementary.pdf]

Table S1. Results of cell and colony morphology, physiological and biochemical tests performed on LAB isolates. Isolates identified from multiple sources such as kefir (K), CWK and/or CWK-fermented sourdough (CWKS) have been labelled accordingly.

**Test**	***Isolate 1*** **(Source-K, CWK, CWKS)**	***Isolate 2*** **(Source-K, CWK, CWKS)**	**Isolate 3 (Source-K, CWK, CWKS)**	***Isolate 4*** **(Source-K, CWK, CWKS)**	***Isolate 5*** **(Source-K, CWK, CWKS)**	***Isolate 6*** **(Source-K, CWK, CWKS)**	***Isolate 7*** **(Source-K, CWK, CWKS)**	***Isolate 8*** **(Source-K, CWK, CWKS)**	***Isolate 9*** **(Source-K, CWK, CWKS)**	***Isolate 10*** **(Source-K, CWK, CWKS)**
Colony colour	Transparent	Transparent	Transparent	Transparent	Transparent	Transparent	Transparent	Transparent	Transparent	Transparent
Surface appearance	Smooth/moist	Smooth/moist	Smooth/moist	Smooth/moist	Smooth/moist	Smooth/moist	Smooth/moist	Smooth/moist	Smooth/moist	Smooth/moist
Cell morphology	Bacilli	Bacilli	Bacilli	Bacilli	Bacilli	Bacilli	Bacilli	Bacilli	Bacilli	Bacilli
Gram reaction	+	+	+	+	+	+	+	+	+	+
Catalase test	−	−	−	−	−	−	−	−	−	−
Spore formation	−	−	−	−	−	−	−	−	−	−
Nitrate reduction test	−	−	−	−	−	−	−	−	−	−
Indole test	−	−	−	−	−	−	−	−	−	−
6.5% NaCl growth test	−	−	−	+	+	+	+	+	+	+
10% NaCl growth test	−	−	−	−	−	−	−	−	−	−
4 °C growth test	−	−	−	−	−	−	−	−	−	−
15 °C growth test	+	+	+	+	+	+	+	+	+	+
45 °C growth test	−	−	−	−	−	−	−	−	−	−
Growth at pH 3	−	−	−	−	−	−	−	−	−	−
Growth at pH 4	−	−	−	−	−	−	−	−	−	−
Growth at pH 5	−	−	−	−	−	−	−	−	−	−
0.1% Bile salt	+	+	+	+	+	+	+	+	+	+
0.9% Bile salts	+	+	+	+	+	+	+	+	+	+
1.5% bile salts	+	+	+	+	+	+	+	+	+	+
2% bile salts	+	+	+	+	+	+	+	+	+	+
Gas production during glucose fermentation in a Durham tube	+	+	+	+	+	+	+	+	+	+
Glycerol	−	−	−	+	+	+	−	−	−	−
D-Ribose	−	−	−	+	+	+	+	+	+	−
D-xylose	+	+	+	−	−	−	−	−	−	−

D-Galactose	+	+	+	+	+	+	+	+	+	+
D-Glucose	+	+	+	+	+	+	+	+	+	+
D-Fructose	+	+	+	+	+	+	+	−	−	+
D-Mannose	+	+	+	+	+	+	+	−	−	−
Mannitol	−	−	−	+	+	+	+	+	+	+
Sorbitol	−	−	−	+	+	+	−	−	−	−
N-acetyl-glucosamine	−	−	−	+	+	+	+	−	−	−
Amygdalin	−	−	−	−	−	−	−	+	+	−
Arbutin	−	−	−	+	+	+	−	+	+	−
Esculin	−	−	−	+	+	+	−	+	+	−
Salicin	−	−	−	+	+	+	+	−	−	−
Cellobiose	−	−	−	+	+	+	−	−	−	−
Maltose	+	+	+	+	+	+	+	+	+	−
Lactose	+	+	+	+	+	+	−	+	+	−
Melibiose	+	+	+	+	+	+	−	+	+	+
Sucrose	+	+	+	+	+	+	+	+	+	+
Trehalose	−	−	−	+	+	+	+	−	−	+
Melezitose	−	−	−	−	−	−	−	−	−	−
D-Raffinose	−	−	−	+	+	+	−	+	+	−
Starch	+	+	+	−	−	−	−	−	−	−
Glycogen	+	+	+	−	−	−	−	−	−	−
D-Gentiobiose	−	−	−	+	+	+	−	−	−	−
D-Turanose	−	−	−	+	+	+	−	−	−	−
D-Lyxose	−	−	−	−	−	−	−	−	−	−
D-Tagatose	−	−	−	+	+	+	−	−	−	−
D-Arabitol	−	−	−	+	+	+	−	−	−	−
Gluconate	+	+	+	+	+	+	−	−	−	+
Fermentation	Obligately Heterofermentative	Obligately Heterofermentative	Obligately Heterofermentative	Facultatively Heterofermentative	Facultatively Heterofermentative	Facultatively Heterofermentative	Obligately Heterofermentative	Obligately Heterofermentative	Obligately Heterofermentative	Obligately Heterofermentative
Isolate identified	***Limosilactobacillus fermentum***	***Limosilactobacillus fermentum***	***Limosilactobacillus fermentum***	***Lactobacillus plantarum***	***Lactobacillus plantarum***	***Lactobacillus plantarum***	***Lactobacillus fusant***	***Lactobacillus reuteri***	***Lactobacillus reuteri***	***Lactobacillus kunkeei***

Table S2. Results of cell and colony morphological, physiological and biochemical tests performed on AAB isolates. Isolates identified from multiple sources such as kefir (K), CWK and/or CWK-fermented sourdough (CWKS) have been labelled accordingly.

**Tests**	***Isolate 1* (Source—K, CWK, CWKS)**	***Isolate 2* (Source- K, CWK, CWKS)**	***Isolate 3* (Source -K, CWK, CWKS)**	***Isolate 4* (Source- K, CWK, CWKS)**	***Isolate 5* (Source-K, CWK, CWKS)**
Colony colour	White and opaque	White and opaque	White and opaque	White and opaque	Cream colour and opaque
Surface appearance	Smooth	Smooth	Smooth	Smooth	Smooth
Cell morphology	Bacilli or oblong	Bacilli or oblong	Bacilli or oblong	Bacilli or oblong	Oblong
Gram reaction test	−	−	−	−	−
Catalase test	+	+	+	+	+
Oxidation of ethanol	+	+	−	−	−
Oxidation of acetic acid	−	−	−	−	−
Methyl red test	−	−	−	−	−
Voges–Proskauer test	−	−	−	−	−
30% glucose growth test	−	−	−	−	−
arginine dihydrolase	−	−	−	−	+
Lysine	−	−	−	−	−
Ornithine decarboxylase	−	−	−	−	−
Citrate Utilisation	−	−	−	−	−
Dihydrogen sulphide	−	−	−	−	−
Carr’s medium	+	+	+	+	+
Urease test	−	−	−	−	+
tryptophane deaminase	−	−	−	−	−
Indole test	−	−	−	−	−
Acetoin	−	−	−	−	+
Fructose	+	+	+	+	+
Ribose	+	+	+	+	+
Xylose	+	+	+	+	+
Galactose	+	+	+	+	+
Mannitol	−	−	−	−	−
Raffinose	−	−	−	−	−
Inositol	−	−	−	−	−
Sorbitol	−	−	−	−	−

Rhamnose	+	+	−	−	−
Sucrose	−	−	−	−	−
Melibiose	+	+	+	+	−
Amygdalin	+	+	+	+	−
Arabinose	+	+	+	+	−
Nitrogen dioxide	−	−	+	+	−
Nitrite	−	−	+	+	−
Isolate identified	***Acetobacter aceti***	***Acetobacter aceti***	***Acetobacter lovaniensis***	***Acetobacter lovaniensis***	***Acetobacter pasteurianus***

Table S3. Results of cell and colony morphology, physiological and biochemical tests performed on yeast isolates. Isolates identified from multiple sources such as kefir (K), CWK and/or CWK-fermented sourdough (CWKS), have been labelled accordingly.

**Test**	***Isolate 1* (Source- K, CWK, CWKS)**	***Isolate 2* (Source- K, CWK, CWKS)**	***Isolate 3* (Source- K, CWK, CWKS)**	***Isolate 4* (Source- K, CWK, CWKS)**	***Isolate 5* (Source- K, CWK, CWKS)**	***Isolate 6* (Source- K, CWK, CWKS)**	***Isolate 7* (Source- K, CWK, CWKS)**
Colony colour	Cream	Pink–red	Milky white	Milky white	Milky white	White	White
Surface appearance	Glossy and smooth	Smooth and glossy	Smooth	Smooth	Smooth	Smooth	Smooth
Cell morphology	Oval	Oval	Oval	Oval	Oval	Oval	Oval
Vegetative propogation	Budding	Budding	Budding	Budding	Budding	Budding	Budding
Mycelium growth test	Pseudohypha	Pseudohypha	Pseudohypha	Pseudohypha	Pseudohypha	Pseudohypha	Pseudohypha
Urease test	−	−	−	−	−	−	−
Nitrate reduction test	−	−	−	−	−	−	−
D-Glucose as a carbon source	+	+	+	+	+	+	+
Glycerol	+	−	−	−	−	+	+
Calcium 2-Keto-Gluconate	−	−	−	−	−	+	−
L-Arabinose	−	−	−	−	−	+	−
D-Xylose as a carbon source	+	−	−	−	−	+	−
Adonitol	−	+	−	−	−	+	−
Xylitol	+	+	−	−	−	+	−
D-Galactose as a carbon source	+	+	+	+	+	+	−
Inositol	−	−	−	−	−	−	−
D-Sorbitol as a carbon source	+	−	−	−	−	+	+
Methyl-α-D-Glucopyranoside	−	−	−	−	−	+	−

N-Acetyl-Glucosamine	−	−	−	−	−	+	−
D-Cellobiose as a carbon source	+	−	−	−	−	+	−
D-Lactose as a carbon source	+	−	−	−	−	−	−
D-Maltose as a carbon source	−	+	+	+	+	+	−
D-Saccharose as a carbon source	+	+	+	+	+	+	+
D-Trehalose as a carbon source	−	−	−	−	−	+	+
D-Melezitose as a carbon source	−	−	−	−	−	+	−
D-Raffinose as a carbon source	+	+	+	+	+	+	+
Isolate identified	***Candida kefyr***	***Rhodotorula mucilaginosa***	***Saccharomyces cerevisiae***	***Saccharomyces cerevisiae***	***Saccharomyces cerevisiae***	***Candida guilliermondii***	***Candida colliculosa***

Table S4. Sanger sequencing of each isolate of the LAB and AAB species.

Identified organisms (% identity match)	Accession number*	E-value*	Maximum score*
Limosilactobacillus fermentum strain CAU6479 16S ribosomal RNA gene, partial sequence (100)	MF582851.1	0.0	2619
Lactobacillus plantarum strain RS66X 16S ribosomal RNA gene, partial sequence (100)	MN450268.1	0.0	2536
Lactobacillus Fusant XU1 16S ribosomal RNA gene, partial sequence (99)	KT335719.1	0.0	2687
Lactobacillus reuteri DSM 20016, 16S ribosomal RNA gene, partial sequence (100)	NR_119069.1	0.0	2883
Lactobacillus kunkeei strain H14_2_1BCO2 16S ribosomal RNA gene, partial sequence (100)	KF599421.1	0.0	2582
Acetobacter aceti strain W1 16S ribosomal RNA gene, partial sequence (100)	KC662508.1	0.0	2676
Acetobacter lovaniensis strain NBRC 13753 16S ribosomal RNA, partial sequence (100)	NR_040832.1	0.0	2665
Acetobacter pasteurianus strain bh12 16S ribosomal RNA gene, partial sequence (100)	FJ227313.1	0.0	2326

The E-value* determines the number of hits one can "expect" to see by chance when searching a database of a particular size, which in this case is 0. Therefore, this implies that there were no random hits in the identification of LAB and AAB species on NCBI BLAST. The accession number* is the unique identification number on the NCBI BLAST database. The maximum score* is the total score that has been identified as that particular microorganism, which is the highest alignment score of a set of aligned segments from the same subject (database) sequence. The percentage of identity was determined by performing multiple sequence alignments in BLAST.

Supplementary File S1. Sanger sequencing output: 16s rDNA partial sequences for LAB and AAB.

LAB Isolate 1: *Limosilactobacillus fermentum* strain CAU6479 16S ribosomal RNA gene.

5′ACTGATTGATGGTGCTTGCACCTGATTGACGATGGATCAC
CAGTGAGTGGCGGACGGGTAGTAACACGTAGGTAACCTGCCCC
GGAGCGGGGGATAACATTTGGAAACAGATGCTAATACCGCATA
ACAACAAAAGCCACATGGCTTTTGTTTGAAAGATGGCTTTGGC
TATCACTCTGGGATGGACCTGCGGTGCATTAGCTAGTTGGTAAG
GTAACGGCTTACCAAGGCGATGATGCATAGCCGAGTTGAGAGA
CTGATCGGCCACAATGGAACTGAGACACGGTCCATACTCCTAC
GGGAGGCAGCAGTAGGGAATCTTCCACAATGGGCGCAAGCCT
GATGGAGCAACACCGCGTGAGTGAAGAAGGGTTTCGGCTCGT
AAAGCTCTGTTGTTGGAGAAGAACGTGCGTGAGAGTAACTGTT
CACGCAGTGACGGTATCCAACCAGAAAGTCACGGCTAACTACG
TGCCAGCAGCCGCGGTAATACGTAGGTGGCAAGCGTTATCCGG
ATTTATTGGGCGTAAAGCGAGCGCAGGCGGTTGCTTAGGTCTG
ATGTGAAAGCCTTCGGCTTAACCGAAGAAGTGCATCGGAAACC
GGGCGACTTGAGTGCAGAAGAGGACAGTGGAACTCCATGTGT
AGCGGTGGAATGCGTAGATATATGGAAGAACACCAGTGGCGAA
GGCGGCTGTCTGGTCTGCAACTGACGCTGAGGCTCGAAAGCAT
GGGTAGCGAACAGGATTAGATACCCTGGTAGTCCATGCCGTAA
ACGATGAGTGCTAGGTGTTGGAGGGTTTCCGCCCTTCAGTGCC
GGAGCTAACGCATTAAGCACTCCGCCTGGGGAGTACGACCGCA

AGGTTGAAACTCAAAGGAATTGACGGGGGCCCGCACAAGCGG
TGGAGCATGTGGTTTAATTCGAAGCTACGCGAAGAACCTTACC
AGGTCTTGACATCTTGCGCAACCTTAGAGATAAGGCGTTCCCTT
CGGGGACGCAATGACAGGTGGTGCATGGTCGTCGTCAGCTCGT
GTCGTGAGATGTTGGGTTAAGTCCCGCAACGAGCGCAACCCTT
GTTACTAGTTGCCAGCATTAAGTTGGGCACTCTAGTGAGACTGC
CGGTGACAAACCGGAGGAAGGTGGGGACGACGTCAGATCATC
ATGCCCCTTATGACCTGGGCTACACACGTGCTACAATGGACGGT
ACAACGAGTCGCAAGCTCGCGAGAGTAAGCTAATCTCTTAAAG
CCGTTCTCAGTTCGGACTGTAGGCTGCAACTCGCCTACACGAA
GTCGGAATCGCTAGAATCGCGGATCAGCATGCCGCGGTGAATA
CGTTCCCGGGCCTTGTACACACCGCCCGTCACACCATGGGAGT
TTGTAACGCCCAAAGTCGGTGGCCTAACCATTAGGAGGGAGCC
3′

LAB Isolate 2: *Lactobacillus plantarum* strain RS66X 16S ribosomal RNA gene, partial sequence.

5′TTACCCCACCGACTTTGGGTGTTACAAACTCTCATGGTGT
GACGGGCGGTGTGTACAAGGCCCGGGAACGTATTCACCGCGGC
ATGCTGATCCGCGATTACTAGCGATTCCGACTTCATGTAGGCGA
GTTGCAGCCTACAATCCGAACTGAGAATGGCTTTAAGAGATTA
GCTTACTCTCGCGAGTTCGCAACTCGTTGTACCATCCATTGTAG
CACGTGTGTAGCCCAGGTCATAAGGGGCATGATGATTTGACGTC
ATCCCCACCTTCCTCCGGTTTGTCACCGGCAGTCTCACCAGAGT
GCCCAACTTAATGCTGGCAACTGATAATAAGGGTTGCGCTCGTT
GCGGGACTTAACCCAACATCTCACGACACGAGCTGACGACAA
CCATGCACCACCTGTATCCATGTCCCCGAAGGGAACGTCTAATC
TCTTAGATTTGCATAGTATGTCAAGACCTGGTAAGGTTCTTCGCG
TAGCTTCGAATTAAACCACATGCTCCACCGCTTGTGCGGGCCCC
CGTCAATTCCTTTGAGTTTCAGCCTTGCGGCCGTACTCCCCAGG
CGGAATGCTTAATGCGTTAGCTGCAGCACTGAAGGGCGGAAAC
CCTCCAACACTTAGCATTCATCGTTTACGGTATGGACTACCAGG
GTATCTAATCCTGTTTGCTACCCATACTTTCGAGCCTCAGCGTCA
GTTACAGACCAGACAGCCGCCTTCGCCACTGGTGTTCTTCCATA
TATCTACGCATTTCACCGCTACACATGGAGTTCCACTGTCCTCTT
CTGCACTCAAGTTTCCCAGTTTCCGATGCACTTCTTCGGTTGAG
CCGAAGGCTTTCACATCAGACTTAAAAAACCGCCTGCGCTCGC
TTTACGCCCAATAAATCCGGACAACGCTTGCCACCTACGTATTA
CCGCGGCTGCTGGCACGTAGTTAGCCGTGGCTTTCTGGTTAAAT
ACCGTCAATACCTGAACAGTTACTCTCAGATATGTTCTTCTTTAA
CAACAGAGTTTTACGAGCCGAAACCCTTCTTCACTCACGCGGC
GTTGCTCCATCAGACTTTCGTCCATTGTGGAAGATTCCCTACTG
CTGCCTCCCGTAGGAGTTTGGGCCGTGTCTCAGTCCCAATGTGG
CCGATTACCCTCTCAGGTCGGCTACGTATCATTGCCATGGTGAG
CCGTTACCCCACCATCTAGCTAATACGCCGCGGGACCATCCAA
AAGTGATAGCCGAAGCCATCTTTCAAACTCGGACCATGCGGTC
CAAGTTGTTATGCGGTATTAGCATCTGTTTCCAGGTGTTATCCCC
CGCTTCTGGGCAGGTTTCCCACGTGTTACTCACCAGTTCGCCAC
TCACTCAAATGTAAATCATGATGCAAGCACCAATCAATACCAG
A 3′

LAB Isolate 3: *Lactobacillus Fusant* XU1 16S ribosomal RNA gene, partial sequence.

5′ATGCAGTCGAACGAGTTCTCGTTGATTGCATCGGTGCTTG
CACCGAGATTCAACATGGAACGAGTGGCGGACGGGTGAGTAA
CACGTGGGTAACCTGCCCTTAAGTGGGGGATAACATTTGGAAA

CAGATGCTAATACCGCATAGATCCAAGAACCGCATGGTTCTTGG
CTGAAAGATGGCGTAAGCTATCGCTTTTGGATGGACCCGCGGC
GTATTGCTAGTTGGTGAGGTAATGGCTCACCAAGGCGATGATAC
GTAGCCGAACTGAGAGGTTGATCGGCCACATTGGGACTGAGAC
ACGGCCCAAACTCCTACGGGAGGCAGCAGTAGGGAATCTTCC
ACAATGGACGCAAGTCTGATGGAGCAACGCCGCGTGAGTGAA
GAAGGCTTTCGGGTCGTAAAACTCTGTTGTTGGAGAAGAATGG
TCGGCAGAGTAACTGTTGTCGGCGTGACGGTATCCAACCAGAA
AGCCACGGCTAACTACGTGCCAGCAGCCGCGGTAAACGTAGGT
GGCAAGCGTTATCCGGATTTATTGGGCGTAAAGCGAGCGCAGG
CGGTTTTTTAAGTCTGATGTGAAAGCCCTCGGCTTAACCGAGGA
AGCGCATCGGAAACTGGGAAACTTGAGTGCAGAAGAGGACAG
TGGAACTCCATGTGTAGCGGTGAAATGCGTAGATATATGGAAG
AACACCAGTGGCGAAGGCGGCTGTCTGGTCTGTAACTGACGCT
GAGGCTCGAAAGCATGGGTAGCGAACAGGATTAGATACCCTGG
TAGTCCATGCCGTAAACGATGAATGCTAGGTGTTGGAGGGTTTC
CGCCCTTCAGTGCCGCAGCTAACGCATTAAGCATTCCGCCTGG
GGAGTACGACCGCAAGGTTGAAACTCAAAGGAATTGACGGGG
GCCCGCACAAGCGGTGGAGCATGTGGTTTAATTCGAAGCAACG
CGAAGAACCTTACCAGGTCTTGACATCTTTTGATCACCTGAGAG
ATCAGGTTTCCCCTTCGGGGGCAAAATGACAGGTGGTGCATGG
TTGTCGTCAGCTCGTGTCGTGAGATGTTGGGTTAAGTCCCGCAA
CGAGCGCAACCCTTATGACTAGTTGCCAGCATTTAGTTGGGCAC
TCTAGTAAGACTGCCGGTGACAAACCGGAGGAAGGTGGGGAT
GACGTCAAATCATCATGCCCCTTATGACCTGGGCTACACACGTG
CTACAATGGATGGTACAACGAGTTGCGAGACCGCGAGGTCAA
GCTAATCTCTTAAAGCCATTCTCAGTTCGGACTGTAGGCTGCAA
CTCGCCTACACGAAGTCGGAATCGCTAGTAATCGCGGATCAGC
ACGCCGCGGTGAATACGTTCCCGGGCCTTGTACACACCGCCCG
TCACACCATGAGAGTTTGTAACACCCGAAGCCGGTGGCGTAAC
TCCTTTAGGGAGCGAGCCGTCTAAGGTGACAAATT3′

LAB Isolate 4: *Lactobacillus reuteri* DSM 20016, 16S ribosomal RNA gene, partial sequence.

5′AGAGTTTGATNNTGGCTCAGGATGAACGCCGGCGGTGTG
CCTAATACATGCAAGTCGTACGCACTGGCCCAACTGATTGATGG
TGCTTGCACCTGATTGACGATGGATCACCAGTGAGTGGCGGAC
GGGTGAGTAACACGTAGGTAACCTGCCCCGGAGCGGGGGATA
ACATTTGGAAACAGATGCTAATACCGCATAACAACAAAAGCCG
CATGGCTTTTGTTTGAAAGATGGCTTTGGCTATCACTCTGGGATG
GACCTGCGGTGCATTAGCTAGTTGGTAAGGTAACGGCTTACCA
AGGCGATGATGCATAGCCGAGTTGAGAGACTGATCGGCCACAA
TGGAACTGAGACACGGTCCATACTCCTACGGGAGGCAGCAGTA
GGGAATCTTCCACAATGGGCGCAAGCCTGATGGAGCAACGCC
GCGTGAGTGAAGAAGGGTTTCGGCTCGTAAAGCTCTGTTGTTG
GAGAAGAACGTGCGTGAGAGTAACTGTTCNCGCAGTGACGGT
ATCCAACCAGAAAGTCACGGCTAACTACGTGCCAGCAGCCGC
GGTAATACGTAGGTGGCAAGCGTTATCCGGATTTATTGGGCGTA
AAGCGAGCGCAGGCGGTTGCTTAGGTCTGATGTGAAAGCCTTC
GGCTTAACCGAAGAAGTGCATCGGAAACCGGGCGACTTGAGT
GCAGAAGAGGACAGTGGAACTCCATGTGTAGCGGTGGAATGC
GTAGATATATGGAAGAACACCAGTGGCGAAGGCGGCTGTCTGG
TCTGCAACTGACGCTGAGGCTCGAAAGCATGGGTAGCGAACA
GGATTAGATACCCTGGTAGTCCATGCCGTAAACGATGAGTGCTA
GGTGTTGGAGGGTTTCCGCCCTTCAGTGCCGGAGCTAACGCATT
AAGCACTCCGCCTGGGGAGTACGACCGCAAGGTTGAAACTCA

AAGGAATTGACGGGGGCCCGCACAAGCGGTGGAGCATGTGGT
TTAATTCGAAGCTACGCGAAGAACCTTACCAGGTCTTG1ACATC
TTGCGCTAACCTTAGAGATAAGGCGTTNCCTTCGGGGACGCAA
TGACAGGTGGTG1CATGGTCGTCGTCAGCTCGTGTCGTGAGATG
TTGGGTTAAGTCCTGCAACGAGCGCAACCCTTGTTACTAGTTGC
CAGCATTAAGTTGGGCACTCTAGTGAGACTGCCGGTGACAAAC
CGGAGGAAGGTGGGGACGACGTCAGATCATCATGCCCCTTATG
ACCTGGGCTACACACGTGCTACAATGGACGGTACAACGAGTCG
CAAGCTCGCGAGATAAGCTAATCTCTTAAAGCCGTTCTCAGTTC
GGACTGTAGGCTGCAACTCGCCTACACGAAGTCGGAATCGCTA
GTAATCGCGGATCAGCATGCCGCGGTGAATACGTTCCCGGGCC
TTGTACACACCGCCCGTCACACCATGGGAGTTTGTAACGCCCA
AAGTCGGTGGCCTAACCTTTATGGAGGGAGCCGCCTAAGGCGG
GACAGATGACTGGGGTGAAGTCGTAACAAGGTAGCCGTAGGA
GAGCCTGCGGCTGGATCACCTCCTTTNT3′

LAB Isolate 5: *Lactobacillus kunkeei* strain H14_2_1 BCO2 16S ribosomal RNA gene, partial sequence.

5′GACGAGCTCTCCTGAATTGATTTTATGCTTGCATAAATGAT
TTTTAGATTCGGAGCGAGTGGCGAACTGGTGAGTAACACGTGG
GTAACCTGCCCCGAAGCGGGGGATAACATTTGGAAACAAATGC
TAATACCGCATAATTAGGTGGAACCGCATGGTTCCAACTTGAAA
GATGGCTCTGCTATCACTTTGGGATGGACCCGCGCCGTATTAGT
TAGTTGGTGAGATAAAAGCCCACCAAGACCATGATACGTAGCC
GACCTGAGAGGGTAATCGGCCACATTGGGACTGAGACACGGC
CCAGACTCCTACGGGAGGCAGCAGTAGGGAATCTTCCACAATG
GACGAAAGTCTGATGGAGCAACGCCGCGTGAGTGATGAAGGT
TTTCGGATCGTAAAACTCTGTTGTTAAAGAAGAACAAGTGTTAG
AGTAACTGTTAACACTTTGACGGTATTTAACCAGAAAGCCACG
GCTAACTACGTGCCAGCAGCCGCGGTAATACGTAGGTGGCAAG
CGTTGTCCGGATTTATTGGGCGTAAAGCGAGCGCAGGCGGATTT
GTAAGTCTGCTGTGAAAGCCCTCAGCTCAACTGAGGAAGTGCA
GTGGAAACTACAAAACTTGAGTACAGAAGAGGAAAGTGGAAC
TCCATGTGTAGCGGTGAAATGCGTAGATATATGGAAGAACACC
AGTGGCGAAGGCGGCTTTCTGGTCTGTTACTGACGCTGAGGCT
CGAAAGCATGGGTAGCGAACAGGATTAGATACCCTGGTAGTCC
ATGCCGTAAACGATGAATACTAGGTGTTGGAGGGTTTCCGCCCT
TCAGTGCCGCAGCTAACGCATTAAGTATTCCGCCTGGGGAGTAC
GACCGCAAGGTTGAAACTCAAAGGAATTGACGGGGGCCCGCA
CAAGTGGTGGAGCATGTGGTTTAATTCGATGCTACGCGAAGAA
CCTTACCAGCTCTTGACATCTTCTGCCAACCCAAGAGATTGGGC
GTTCCCTTCGGGGACAGAATGACAGGTGGTGCATGGTTGTCGT
CAGCTCGTGTCGTGAGATGTTGGGTTAAGTCCCGCAACGAGCG
CAACCCTTATTATTAGTTGCCAGCATTTAGTTGGGCACTCTAGTG
AGACTGCCGGTGATAAACCGGAGGAAGGTGGGGACGACGTCA
AATCATCATGCCCCTTATGAGCTGGGCTACACACGTGCTACAAT
GGATGGTACAACGAGTCGCGAAACCGCGAGGTCAAGCTAATCT
CTTAAAGCCATTCTCAGTTCGGATTGCAGGCTGCAACTCGCCTG
CATGAAGTTGGAATCACTAGTAATCGTGGATCAGCATGCCACG
GTGAATACGTTCCCGGGCCTTGTACACACCGCCCGTCACACCAT
GAGAGTTTGTAACACCCAAAGACGATGGGGTAA3′

AAB Isolate 1: *Acetobacter aceti* strain W1 16S ribosomal RNA gene, partial sequence.

5′AGAGTTTGATTCTGGCTCAGAGCGAACGCTGGCGGCATG
CTTAACACATGCAAGTCGCACGAAGGCTTCGGCCTTAGTGGCG
GACGGGTGAGTAACGCGTAGGAATCTATCCATGGGTGGGGGAT

AACTCCGGGAACTGGAGCTAATACCGCATGATACCTGAGGGTC
AAAGGCGCAAGTCGCCTGTGGAGGAGTCTGCGTTTGATTAGCT
TGTTGGTGGGGTAAAGGCCTACCAAGGCGATGATCAATAGCTG
GTCTGAGAGGATGATCAGCCACACTGGGACTGAGACACGGCC
CAGACTCCTACGGGAGGCAGCAGTGGGGAATATTGGACAATGG
GGGCAACCCTGATCCAGCAATGCCGCGTGTGTGAAGAAGGTTT
TCGGATTGTAAAGCACTTTCGGCGGGGACGATGATGACGGTAC
CCGCAGAAGAAGCCCCGGCTAACTTCGTGCCAGCAGCCGCGG
TAATACGAAGGGGGCTAGCGTTGCTCGGAATGACTGGGCGTAA
AGGGCGTGTAGGCGGTTTGTACAGTCAGATGTGAAATCCCCGG
GCTTAACCTGGGAGCTGCATTTGATACGTGCAGACTAGAGTATG
AGAGAGGGTTGTGGAATTCTCAGTGTAGAGGTGAAATTCGTAG
ATATTGGGAAGAACACCGGTGGCGAAGGCGGCAACCTGGCTC
ATTACTGACGCTGAGGCGCGAAAGCGTGGGGAGCAAACAGGA
TTAGATACCCTGGTAGTCCACGCTGTAAACGATGTGTGCTGGAT
GTTGGGTAACTTAGTTACTCAGTGTCGTAGCTAACGCGATAAGC
ACACCGCCTGGGGAGTACGGCCGCAAGGTTGAAACTCAAAGG
AATTGACGGGGGCCCGCACAAGCGGTGGAGCATGTGGTTTAAT
TCGAAGCAACGCGCAGAACCTTACCAGGGCTTGTATGGAGAG
GCTGTATTCAGAGATGGATATTTCCCGCAAGGGACCTCTTGCAC
AGGTGCTGCATGGCTGTCGTCAGCTCGTGTCGTGAGATGTTGGG
TTAAGTCCCGCAACGAGCGCAACCCTTATCTTTAGTTGCCAGCA
TGTTTGGGTGGGCACTCTAAAGAGACTGCCGGTGACAAGCCGG
AGGAAGGTGGGGATGACGTCAAGTCCTCATGGCCCTTATGTCC
TGGGCTACACACGTGCTACAATGGCGGTGACAGTGGGAAGCTA
GATGGCGACATCGTGCCGATCTCTAAAAACCGTCTCAGTTCGG
ATTGCACTCTGCAACTCGAGTGCATGAAGGTGGAATCGCTAGT
AATCGCGGATCAGCATGCCGCGGTGAATACGTTCCCGGGCCTT
GTACACACCGCCCGTCACACCATGGGAGTTGGTTTGACCTTAA
GCCGGTGAGCGAACCGCAAGGACGCAGCCGACCACGGTCGGG
TCAGCGACTGGGGTGAAGTCGTAACAAGGTAGCC3′

AAB Isolate 2: ***Acetobacter lovaniensis*** **strain NBRC 13753 16S ribosomal RNA, partial sequence.**

5′TGAGTTTTGATCCTGGCTCAGAGCGAACGCTGGCGGCAT
GCTTAACACATGCAAGTCGCACGAACCTTTCGGGGTTAGTGGC
GGACGGGTGAGTAACGCGTAGGAATCTGTCCACGGGTGGGGG
ATAACTCTGGGAAACTGGAGCTAATACCGCATGATACCTGAGG
GTCAAAGGCGCAAGTCGCCTGTGGAGGAGCCTGCGTTCGATTA
GCTAGTTGGTGGGGTAAAGGCCTACCAAGGCGATGATCGATAG
CTGGTTTGAGAGGATGATCAGCCACACTGGGACTGAGACACGG
CCCAGACTCCTACGGGAGGCAGCAGTGGGGAATATTGGACAAT
GGGGGCAACCCTGATCCAGCAATGCCGCGTGTGTGAAGAAGG
TCTTCGGATTGTAAAGCACTTTCGACGGGGACGATGATGACGG
TACCCGTAGAAGAAGCCCCGGCTAACTTCGTGCCAGCAGCCGC
GGTAATACGAAGGGGGCTAGCGTTGCTCGGAATGACTGGGCGT
AAAGGGCGTGTAGGCGGTTTACACAGTCAGATGTGAAATCCCC
GGGCTTAACCTGGGAGCTGCATTTGATACGTGTAGACTAGAGTG
TGAGAGAGGGTTGTGGAATTCCCAGTGTAGAGGTGAAATTCGT
AGATATTGGGAAGAACACCGGTGGCGAAGGCGGCAACCTGGC
TCATGACTGACGCTGAGGCGCGAAAGCGTGGGGAGCAAACAG
GATTAGATACCCTGGTAGTCCACGCTGTAAACGATGTGTGCTAG
ATGTTGGGTAACTTTGTTATTCAGTGTCGCAGTTAACGCGTTAA
GCACACCGCCTGGGGAGTACGGCCGCAAGGTTGAAACTCAAA
GGAATTGACGGGGGCCCGCACAAGCGGTGGAGCATGTGGTTTA
ATTCGAAGCAACGCGCAGAACCTTACCAGGGCTTGAATGTAGA

GGCTGTATTCAGAGATGGATATTTCCCGCAAGGGACCTCTAACA
CAGGTGCTGCATGGCTGTCGTCAGCTCGTGTCGTGAGATGTTGG
GTTAAGTCCCGCAACGAGCGCAACCCCTATCTTTAGTTGCCAGC
ATGTTTGGGTGGGCACTCTAGAGAGACTGCCGGTGACAAGCCG
GAGGAAGGTGGGGATGACGTCAAGTCCTCATGGCCCTTATGTC
CTGGGCTACACACGTGCTACAATGGCGGTGACAGTGGGAAGCT
AGATGGTGACATCATGCTGATCTCTAAAAGCCGTCTCAGTTCGG
ATTGCACTCTGCAACTCGAGTGCATGAAGGTGGAATCGCTAGT
AATCGCGGATCAGCATGCCGCGGTGAATACGTTCCCGGGCCTT
GTACACACCGCCCGTCACACCATGGGAGTTGGTTTGACCTTAA
GCCGGTGAGCGAACCCGCAAGGGGCGCAGCCGACCACGGTCG
GGTCCAGCGACTGGGGTGAAGTCGTAC3′

AAB Isolate 3: *Acetobacter pasteurianus* strain bh12 16S ribosomal RNA gene, partial sequence.

5′CCAATGGCGGCAGCTTACACATGCAGTCGCACGAAGGTT
TCGGCCTTAGTGGCGGACGGGTGAGTAACGCGTAGGTATCTATC
CATGGGTGGGGGATAACACTGGGAAACTGGTGCTAATACCGCA
TGACACCTGAGGGTCAAAGGCGCAAGTCGCCTGTGGAGGAGC
CTGCGTTTGATTAGCTAGTTGGTGGGGTAAAGGCCTACCAAGGC
GATGATCAATAGCTGGTTTGAGAGGATGATCAGCCACACTGGG
ACTGAGACACGGCCCAGACTCCTACGGGAGGCAGCAGTGGGG
AATATTGGACAATGGGGGCAACCCTGATCCAGCAATGCCGCGT
GTGTGAAGAAGGTCTTCGGATTGTAAAGCACTTTCGACGGGGA
CGATGATGACGGTACCCGTAGAAGAAGCCCCGGCTAACTTCGT
GCCAGCAGCCGCGGTAATACGAAGGGGGCTAGCGTTGCTCGG
AATGACTGGGCGTAAAGGGCGTGTAGGCGGTTTGTACAGTCAG
ATGTGAAATCCCCGGGCTTAACCTGGGAGCTGCATTTGATACGT
GCAGACTAGAGTGTGAGAGAGGGTTGTGGAATTCCCAGTGTAG
AGGTGAAATTCGTAGATATTGGGAAGAACACCGGTGGCGAAG
GCGGCAACCTGGCTCATTACTGACGCTGAGGCGCGAAAGCGTG
GGGAGCGGACAGGATTAGATACCCTGGTAGTCCACGCTGTAAA
CGATGTGTGCTAGATGTTGGGTGACTTAGTCATTCAGTGTCGCA
GTTAACGCGTTAAGCACACCGCCTGGGGAGTACGGCCGCGA
GGTTGAAACTCAAAGGAATTGACGGGGGCGCCGCACAAGCG
GTGGAGCATGTGGTTGAATTCGAAAGCAACGCGCAGAACCTTA
CCACGGCTTGGAGTGTAGAGGCTGCAAGCAGAGATGTTTGTTT
CCCGCAAGGGACCTCTAACACAGGTGCTGGCGTGGCTGTCGTC
AGCTCGTGTCGTGAGATGTTGGGTTAAGTCCCTCAGCGAGCGC
AACCCGCTATCTTTAGTTGCCATCAAGTTTGGCCTGGGCACTCT
AGGAGAGACTGCCAGGTGACCGAGCCCCGCACAAGGTGGGAG
AATGACGTGAAGTCCTCATGGCCGCTTAAGGGTGCCGTGGGAC
ACGTGCTACAATGGCGGTGACAGTGGGAAGCTAGGTGGTGAC
ACCATGCTGATCTCTAAAAGCCGTCTCAGTTCGGATTGCACTCT
GCAACTCGAGTGCATGAAGGTGGAATCGCTAGTAATCGCGGAT
CAGCATGCCGCGGTGAATACGTTCCCGGGCCTTGTACACACCG
CCCGTCACACCATGGGAGTTGGTTTGACCTTAAGCCGGTGAGC
GAACCGCAAGGACGCAGCCGACCACGTCGTACGCGT3′
